# Supplementary material for: Venomix: a simple bioinformatic pipeline for identifying and characterizing toxin gene candidates from transcriptomic data
Source: PeerJ. 2018 Jul 31;6:e5361. doi: 10.7717/peerj.5361 (PMC6074769; doi:10.7717/peerj.5361)
Supplement: Supplemental Information 6 [file peerj-06-5361-s006.gz › FinalOutput_1E-20/Thrombin-like_enzyme_ancrod_11/finaltree.pdf]

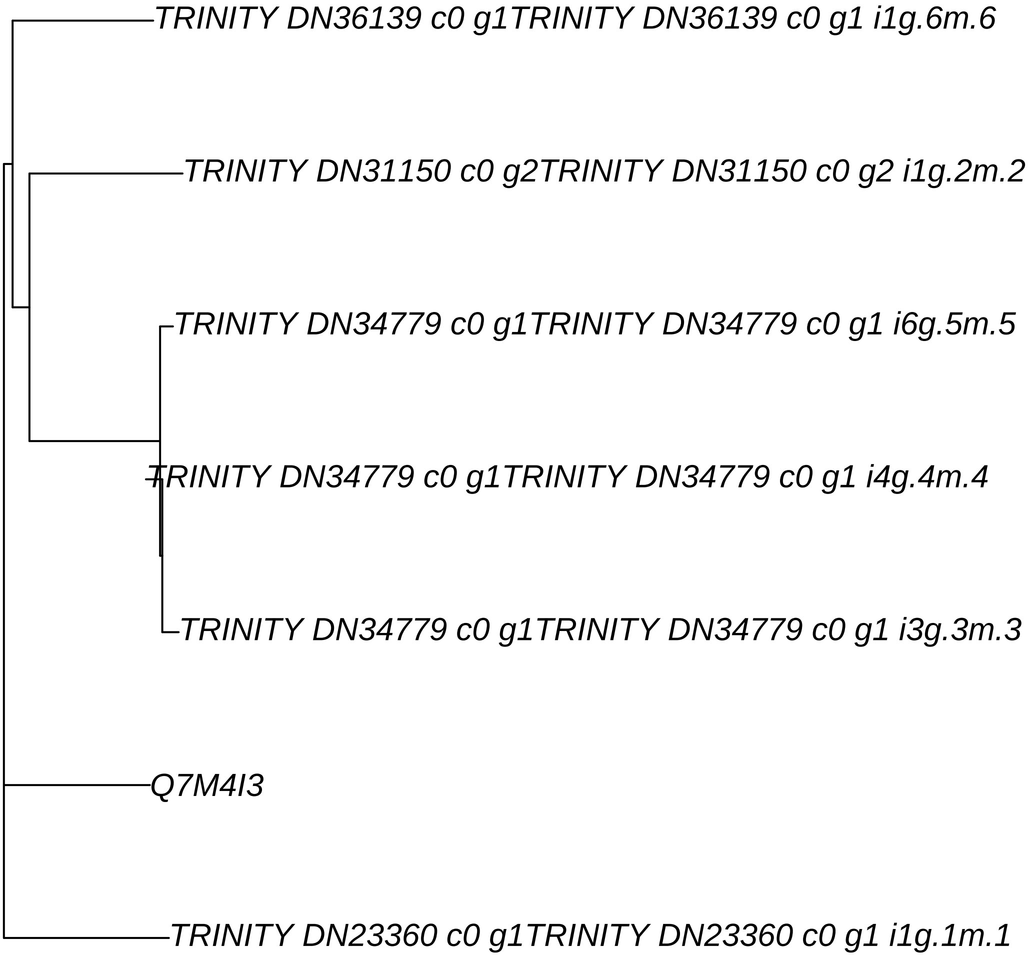
TRINITY DN36139 c0 g1TRINITY DN36139 c0 g1 i1g.6m.6
TRINITY DN31150 c0 g2TRINITY DN31150 c0 g2 i1g.2m.2
TRINITY DN34779 c0 g1TRINITY DN34779 c0 g1 i6g.5m.5
TRINITY DN34779 c0 g1TRINITY DN34779 c0 g1 i4g.4m.4
TRINITY DN34779 c0 g1TRINITY DN34779 c0 g1 i3g.3m.3
Q7M4I3
TRINITY DN23360 c0 g1TRINITY DN23360 c0 g1 i1g.1m.1
